# Ear Reconstruction Simulation: From Handcrafting to 3D Printing

**DOI:** 10.3390/bioengineering6010014

**Published:** 2019-02-05

**Authors:** Elisa Mussi, Rocco Furferi, Yary Volpe, Flavio Facchini, Kathleen S. McGreevy, Francesca Uccheddu

**Affiliations:** 1 Department of Industrial Engineering, University of Florence, via di Santa Marta, 3, 50139 Firenze, Italy; rocco.furferi@unifi.it (R.F.); yary.volpe@unifi.it (Y.V.); francesca.uccheddu@unifi.it (F.U.); 2 Department of Plastic Surgery, Meyer Children’s Hospital, Viale Gaetano Pieraccini, 24, 50139 Firenze; Italy; flavio.facchini@meyer.it; 3 Office of International Relations and the Promotion of Innovation, Meyer Children’s Hospital, Viale Gaetano Pieraccini, 24, 50139 Firenze, Italy; kathleen.mcgreevy@meyer.it

**Keywords:** Computer-Aided Design (CAD), additive manufacturing, microtia, autologous ear reconstruction, simulation, training, image-processing, costal cartilage, silicone rubbers

## Abstract

Microtia is a congenital malformation affecting one in 5000 individuals and is characterized by physical deformity or absence of the outer ear. Nowadays, surgical reconstruction with autologous tissue is the most common clinical practice. The procedure requires a high level of manual and artistic techniques of a surgeon in carving and sculpting of harvested costal cartilage of the patient to recreate an auricular framework to insert within a skin pocket obtained at the malformed ear region. The aesthetic outcomes of the surgery are highly dependent on the experience of the surgeon performing the surgery. For this reason, surgeons need simulators to acquire adequate technical skills out of the surgery room without compromising the aesthetic appearance of the patient. The current paper aims to describe and analyze the different materials and methods adopted during the history of autologous ear reconstruction (AER) simulation to train surgeons by practice on geometrically and mechanically accurate physical replicas. Recent advances in 3D modelling software and manufacturing technologies to increase the effectiveness of AER simulators are particularly described to provide more recent outcomes.

## 1. Introduction

Craniofacial microsomia (CFM) is a term to describe a spectrum of malformations mainly affecting the anatomical regions that developes from first and second branchial arches during the 5th–6th weeks of gestation [1]. People with CFM usually have ear abnormalities affecting one or both ears. A common congenital malformation is the so called microtia, i.e., the presence of an underveloped external ear. In fact, microtia has a worldwide prevalence of 2.06 per 10,000 births [2], with a higher incidence in Asian, Hispanic, and Native American populations with a prevalence in males [3]. Furthermore, microtia affects the right ear twice as often as the left.

**Figure 1:**
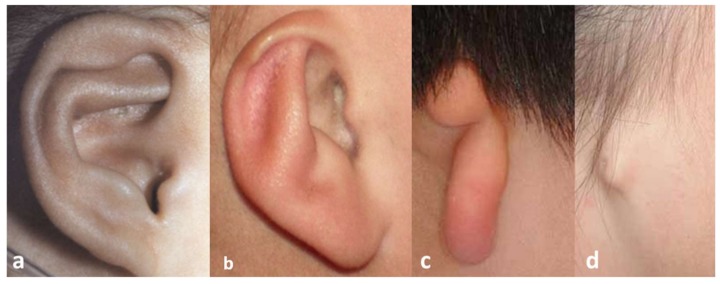
Grade of microtia: (**a**) grade I, (**b**) grade II, (**c**) grade III, (**d**) grade IV.

The malformation can concern size, orientation, shape, and position of the external ear [3] that can degenerate towards the total absence of the ear auricle. In grade I microtia severity, the ear size is slightly small and each of its structures are clearly distinguishable (Figure 1a). In grade II microtia, the size of one ear is one half to two-thirds compared with the physiological sizes and helix or lobule of ear, and the auricle can be absent (Figure 1b). Grade III microtia is the most common type of microtia and is characterized by a “peanut” shape of the ear (Figure 1c) [4]. Finally, in grade IV microtia, commonly named anotia, the external ear is completely absent (Figure 1d).

Microtia is usually accompanied by aural atresia or stenosis with conductive hearing loss (80% of cases) [5]. In children, anotia/microtia can be linked to speech and language delays, learning difficulty at school [6], and difficulties in human interactions [7]. Malformation and absence of an ear can also implicate negative psychological effects due to aesthetic modification of the face, lack of symmetry, and differences in the appearance of the ears [8], as well as functional problems such as, for instance, in wearing glasses. Not by chance, lack of confidence, dissatisfaction, and depression have been reported in 55% of microtia patients, and signs of anxiety have been shown in 52% of subjects [9] compromising the quality of their life.

Prostheses and surgical approaches are used to reshape the ear malformation reducing both physical and psychological issues and improving health-related quality of life [10].

The microtia can be treated through three different clinical approaches: (i) silicone ear prostheses fixed through osseointegrated implants or adhesive (prosthetic approach), (ii) auricular reconstruction with synthetic material implant, (iii) auricular reconstruction with autologous tissue (costal cartilage harvested from the patient) [11].

The prosthetic approach (i) is a non-permanent solution that involves the use of a silicone artificial ear attached to the patient through craniofacial bones anchored implants or medical grade skin adhesives [12]. Such an approach is a suitable solution for patients who are unable to undergo surgery since it is less invasive and there is less risk of infection [3]. Generally, the ear prosthesis is made with room-temperature vulcanizing (RTV) silicone rubbers able to simulate several skin characteristic such as pigmentation, hardness, and tensile strength [13]. Traditionally, ear prostheses are produced by technicians through a process that involves an impression of the healthy contralateral ear to be used as a model for mirroring and wax sculpting of the future prosthesis. The mold is fabricated and silicone is injected with color into the mold to get the prosthesis.

This method presents several challenges: it is time consuming and requires good sculpting and carving skills that closely influence the outcomes’ quality. To overcome such limitations, thus allowing the surgeons to achieve better results in terms of product quality, costs and production time when compared with traditional manual techniques, Computer-Aided Design/Manufacturing (CAD/CAM) technologies and additive manufacturing have been studied in the last decade. In most of the scientific works proposed in the literature [13,14,15,16,17,18,19,20], the process of prosthesis production foresees the subsequent phases:

1. Acquisition of a 3D structure of the ear by means of CT scan, photogrammetry or 3D scanner;
2. 3D modelling of the prosthesis using CAD/CAM tools;
3. Prosthesis mold manufacturing through 3D printers;
4. Filling of the mold to obtain the silicone ear prosthesis.

The alloplastic approach (ii) is a permanent solution which is part of the microtia surgical treatment and which uses alloplastic (i.e., synthetic material) implants as support structures for total auricular reconstruction. This alternative surgical technique foresees the subcutaneous implantation of standard (i.e., non-patient specific) ear structures, showing a reduced surgical time, standardizing outcomes among surgeons, and providing a good size match with the contralateral ear and a cosmetically appealing reconstruction [21,22,23,24]. In 1966, Cronin [25] described the use of a Silastic® framework (silicone rubber) as an alloplastic material; the initial outcomes were satisfying, but long-term follow-up highlighted a high percentage of infection and extrusion. For this reason, in 1983 the use of high-density porous polyethylene (Medpor®) was introduced; it is a stable, inert, non-reabsorbable, biocompatible material that, thanks to its porosity promoting tissue ingrowth, can be easily integrated with human tissue [26,27].

Despite the improvement of prosthetic and alloplastic approaches, the use of autologous costal cartilage grafts remains the standard clinical practice for ear reconstruction (iii). The implant longevity as well as the high degree of integration are the main reasons why it is the standard clinical treatment. This procedure, named autologous ear reconstruction (AER), consists of harvesting costal cartilages from the patient and in carving them with a three-dimensional ear shape to implant beneath the skin in the auricular region. More specifically, the framework of the reconstructed ear is held together by several sutures and is inserted into a skin pocket; a surgeon must be careful to position it symmetrically with respect to the contralateral ear to give an aesthetic appearance as natural as possible [28]. The results of the surgery are highly dependent on the *artistic* and technical skills of the clinician and on the quality of the framework replicating the three-dimensional architecture of the ear. The creation of an adequate framework is a difficult process considering the complexity of the geometry to be reproduced, the complexity of the cartilaginous framework and its intimate relationship with soft tissue envelopes [29]. Furthermore, several solutions of surgery can be adopted by the surgeons depending on age, grade of microtia, quality of the skin, and of the cartilages of the patient [28], for which clinical case analysis and preoperative planning are essential. For such reasons, autogenous ear reconstruction is considered a major challenge among reconstructive surgeons which requires practice and a long experience. Within this context, simulation is recognized as a useful tool for reconstructive surgeons, as it allows them to acquire both experience and technical skills in ear reconstruction by simulating interventions, and thus ensuring the quality of results to patients.

In addition to the clinical approaches used to date, in the future, the area of tissue engineering could propose new treatment methods that could become part of the common clinical practice. To date, numerous attempts have been made to create tissue-engineered ear-shaped cartilage that mimic human ear tissue from the point of view of shape, size, biomechanics, and histology using patient-derived or donor cells. The tissue-engineered constructs have been extensively studied in animal models, but several challenges still need to be addressed [30,31,32]. Bioprinting technologies can overcome some of these challenges thanks to the ability to obtain the complex patient-specific ear shapes by depositing biomaterials layer-by-layer in a controllable manner [33]. A first implant attempt of tissue-engineered ear-shaped cartilage on a human subject was performed by Zhou et al. [34] and presented in 2018. It represents a significant breakthrough in the tissue engineering area; however, several limitations, such as optimal biomaterials and the optimal source of sufficient autologous chondrogenic cells mean that 3D-printed auricular cartilage models are still far from being used in common clinical implementation.

This paper will focus on the autologous surgical approach of ear reconstruction with particular attention to the simulation of surgery as a training tool and preoperative planning. More specifically, the present work investigates and analyzes the evolution of materials and methods used in the history of AER simulation from the early use of hand-crafted fresh fruits and vegetables to model the ear to the more recent adoption of synthetic materials (such as silicone rubbers) combined with 3D modelling and printing technologies. Firstly, the importance of medical simulation in surgery as an education tool and preoperative planning aid, with focus on AER will be introduced. Afterwards, an overview of different materials adopted during the history of ear reconstruction training, from the use of vegetables to materials such as silicone, will be presented. The methods used to create the most advanced trainers and devices designed to improve the results of the surgery will be finally explained.

## 2. Simulation and Preoperative Planning

The use of medical simulation for both training and preoperative planning purposes is increasing in actual hospital clinical practice. The traditional approach of residents’ medical training, according to the apprentice-style model of "see one, do one, teach one", implies the acquisition of the clinical skills directly on the patient. The adoption of this learning method exposes the patient to the inexperience of the residents, to potential errors and harms with consequent costs for the health system. In 2016, a study reported that in the US, it is estimated that one death in three is due to medical errors [35], which results in a health care system expenditure of about $19 billion per year [36]. The use of simulation as a training tool is crucial to reduce the risk of medical errors thanks to the possibility of repeating clinical procedures, increasing precision, and improving skills. Furthermore, simulation training allows to overcome some ethical debates on the use of corpses, animals or human subjects for learning, while ensuring effectiveness and safety of patient care [37].

On the other hand, simulation has gradually become a useful clinical tool for the detailed evaluation and training of patient specific clinical cases. Medical simulation often results in a physical reproduction of the patient anatomy; such three-dimensional replicas allow the surgeon to plan the intervention with extreme accuracy, anticipating risks and challenges that may occur. In this way, before the surgery, the physician can study the best intervention strategy often identifying unconventional surgical procedures and access. The physical manipulation of such models has the great advantage of providing the physician with the ability to observe the spatial relationship of sites of interest ensuring that the surgeon familiarizes with the anatomy and creates mental maps to be used in the surgery room.

Within this context, digital 3D scanning, 3D modelling, and 3D printing are crucial tools to the fabrication of learning simulators and 3D anatomic replicas to be employed during surgery. In the last decades, these technologies have started to be widely used in the medical field, revolutionizing clinical practice and surgery.

An effective simulator must provide the surgeon with an accurate anatomic scenario that eases and leads him during the simulation.

The simulator fabrication process is performed in three main steps: the acquisition of patient data (e.g., medical imaging and 3D body scanner), the computer-based modelling of 3D data, and the additive manufacturing of tissues or molds of them.

Diagnostic imaging techniques and 3D scanner technologies [29] can be employed to capture the anatomy of the region of the interest (ROI). More specifically, computed tomography (CT) or magnetic resonance imaging (MRI) are widely employed to acquire internal biological structures to observe, study, and replicate them. Without regard to imaging modality, acquired data are saved as images slices in the DICOM format (digital imaging and communications in medicine) and require to be segmented to build a 3D reconstruction of certain anatomical areas. Segmentation consists in the isolation of the ROI through an automatic or semi-automatic process based on simple region growing, thresholding or clustering algorithms.

On the other hand, 3D scanning enables the direct acquisition of three-dimensional data of the outer anatomical surface without the use of ionizing radiation, non-invasively, and at low cost [38]. In the medical field, several scientific studies report the use of 3D scanners to acquire anatomical data for the construction of orthoses [39] or medical devices [40], but also for diagnostic purposes [41] or maxillofacial reconstruction planning [41,42,43,44].

The patient anatomical data obtained from the 3D scan (or from the segmentation of medical imaging) must firstly imported into a 3D CAD modelling software. Then the data are processed by eliminating artefacts, filling holes, etc., and creating 3D models (in STL format), to be printed directly in plastic materials (e.g., ABS or PLA) or designing and printing molds that are subsequently filled with silicones or gels.

Additive manufacturing (AM) enables the production of 3D objects by the subsequent deposition of material layers (layer-by-layer) from a CAD 3D model. Techniques of AM have many advantages: reduced cost and time, reduction of waste materials, possibility to produce custom devices with complex shapes that are geometrically difficult to achieve with traditional production techniques. Medical professionals have realized the great potential that additive manufacturing has in medicine and its cost efficiency, and this has led to a significant use of AM in health care and medicine for several applications: tissue engineering, regenerative medicine, simulation, surgery [45,46].

In this context, AER does not make an exception. The role of physical simulation (for instance using additive manufactured models) is becoming the new standard for the most advanced hospitals and research centers. In fact, as mentioned in the introductory section, AER is a very complex procedure since it requires a good understanding of the 3-dimensional architecture of the ear and learning the step-by-step construction of a harmonious framework, that includes eminences and depressions formed by the outer helical rim, Y-shaped antihelix, concha bowl, tragus, and antitragus [47]. For this reason, the possibility of simulating the AER would allow the evaluation of the results obtained at each performance and the identification of possible improvements. In other words, training using physical models proves to be a valid instrument to allow surgeons the practice of the reconstruction procedure and at the same time to guarantee the quality and safety of patient care reducing the risk of mistakes and improving the clinical outcomes of AER [48,49].

Furthermore, even the most experienced surgeons need to plan the surgery on the specific anatomical replicas of the patient because each ear is unique, the amount and shape of the costal cartilages are unique, the possible malformations are many, and therefore the surgical approach, the procedure of cutting and modelling the framework, is different for each patient. Consequently, a specific and detailed plan is required to obtain an excellent balance between the expected results and the actual ones.

The major difficulties of the auricular reconstruction procedure lie in the free hand carving and shaping of an auricular framework from the autogenous costal cartilage and in the definition of the minimum amount of cartilage required to obtain the reconstructed ear.

Summing up, the objective of an AER simulator is to train the surgeon:

- To study, evaluate the available costal cartilage of the patient, identify the best cartilage cutting strategy, and optimize the amount of cartilage taken reducing the donor site morbidity;
- To cut and carve costal cartilage to recreate a three-dimensional framework mimicking the curves and shape of a normal ear, giving it an aesthetical natural appear.

Therefore, the fundamental aspects to be considered for the realization of an effective AER simulator are:

- To find the materials whose mechanical properties are similar to the ones of cartilages, and which could be shaped as the actual costal cartilages. This allows the surgeon to train on cutting, modelling, and carving in a realistic way;
- To find a fabrication method for the creation of anatomical replicas of both the costal cartilage and the ear to be used as reference to reconstruct the 3D framework.

Both these two aspects have been explored in the scientific literature, and are described in the following sections.

## 3. Costal Cartilage Simulator Materials: from Potatoes to Silicone

**Figure 2:**
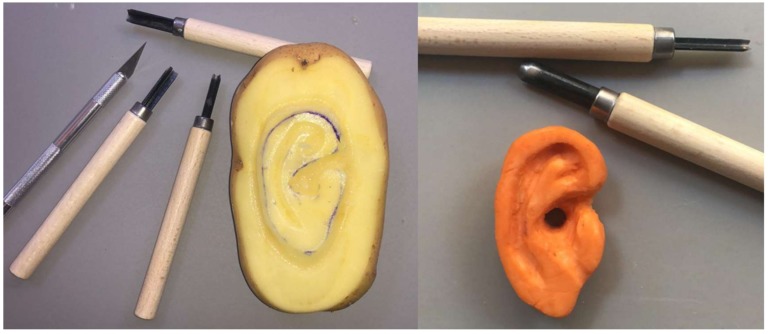
Simulation of ear reconstruction with vegetables.

In literature, a wide range of materials have been used to create costal cartilage models with the aim to help beginners and experts in simulating the AER procedure. The use of a soap bar (price about 1.20 €) to model the ear was the simplest option used in the past (around the 1970s) [50]. Even if this solution is simple, easily available, and low-cost, it is no longer adopted because it is not able to simulate the cartilage neither from the point of view of the mechanical properties nor of the shape one. For this reason, in the early 2000s, vegetables and fruits (price about 0.20 €) such as carrots, apples, and potatoes were used as training model materials (Figure 2).

The pioneer of this approach was Brent [51] who demonstrated the feasibility of modelling ear structures using potatoes and carrots in 2003 during a workshop.

Vadodaria et al. [52] reported that mechanical properties of sweet potatoes such as consistency, elasticity, and flexibility are very similar to costal cartilage (Figure 2), while Agrawal in Reference [53] argues that vegetables are harder to carve, brittle, inelastic, and relatively stiff. It is worth noting that the vegetable solution has the great disadvantage of not being able to replicate the shape and the amount of cartilage available in a human subject. Therefore, vegetable-based simulators allow the physician to practice in modelling but not in the evaluation of the best cutting strategy of the anatomical elements (helix, antihelix, tragus, and antitragus).

To partially overcome such a drawback, ears modelled using ox scapular cartilages were tested by 22 post-graduate trainees in India [53], while Shin and Hong [54] proposed the use of porcine cartilages (price about 5.00 €). In both cases, consistency and flexibility of cartilages were considered satisfactorily mimicking the mechanical properties of human subjects. However, the use of animal cartilage requires compliance with specific storage conditions, safety precautions for the risk of disease transmission, and can lead to ethical problems connected to the use of animals for medical education.

Fresh fruits, vegetables, and animal cartilages are easily available and low-cost solutions to practice and allow to evaluate the surgeon’s manual skills improvements obtained with practice. However, all these solutions are not able to provide the surgeons with a reliable shape of human costal cartilage, thus limiting the realism of the simulation to only replicate the mechanical feeling of the procedure.

Accordingly, the cartilages of human cadavers (2000 €) have been also proposed for training purposes [55]; these, in fact, are certainly characterized by a shape that accurately reflects the real anatomy of patients. However, the use of human cadavers has several negative aspects: the risk of disease transmission, the low availability on the market, and the high cost.

Due to the abovementioned drawbacks, the subsequent step in the research of materials suitable for simulation tasks moved towards the study of synthetic materials focusing on plastic eraser, acrylic polyurethane, and silicone to accomplish the aims of replicating the cartilage shape and properties.

Thadani et al. [56] and Magritz et al. [48] used dental impression silicone to create a model of costal cartilages. The authors reported that the material is characterized by a consistency similar to the human rib cartilage.

**Figure 3:**
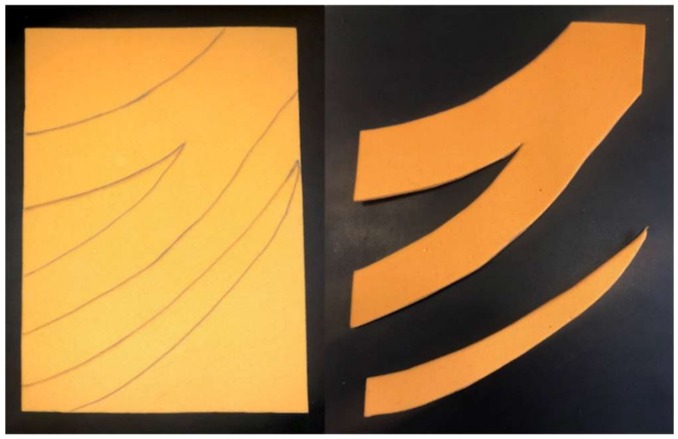
Costal cartilage in plastic eraser.

In 2018, Erdogan et al. [57] proposed the use of plastic eraser (price 4.00 €) made from polyvinyl chloride to create a costal cartilage model (Figure 3). The material is cost-effective and has a texture very similar to the one of human cartilage. The costal cartilage shape was obtained by cutting it from an A6-sized plastic eraser. According to the trainees, the physical replica using plastic eraser has allowed a realistic simulation of the carving and modelling of the cartilages. Nevertheless, the material is not suitable for the use of nylon sutures usually employed to assemble individual anatomical elements of the ear framework; in fact, the material is less elastic and more fragile than the costal cartilage. Furthermore, the shape of the costal cartilage was obtained manually by cutting it from a block of plastic rubber; therefore, the replica does not faithfully reproduce neither the geometry of human cartilage nor the specific geometry of a patient.

A group of Indian clinicians [58] created a 3D model of the costal cartilage of a 7-year-old child using a variant of polyamide and starch, with the aim of assessing the best option for the harvest of the individual anatomical parts for the ear framework construction. The employment of such material guarantees the flexibility of the reproduced cartilage like real ones and at a reasonably low cost.

The employment of training models in synthetic materials described above allows to overcome some issues such as the risk of diseases transmission, the low availability, ethical problems, etc. However, the reproduced geometries of costal cartilages were obtained manually without starting from a real anatomical data.

In 2009, Yamada et al. used dental impression silicone to create a realistic and precise copy of cartilage [59]. They took a negative impression of harvested rib cartilage during real surgery, and then they poured silicone dental material into the negative creating a positive copy of cartilages. In this way, a precise copy of rib cartilage was fabricated, highly faithful to the human anatomical geometries. The authors reported that the replica allowed a very realistic ear reconstruction by using the actual surgery instruments and it was reported to be useful to practice with before surgery.

In 2011, a replica of the costal cartilage in acrylic polyurethane was proposed [60]. The material has a cutting and sculpting behavior similar to that of real cartilage, and therefore it is suitable for a realistic simulation. Furthermore, the cost of such a material is very inexpensive (1.00 €).

Berens et al. [61] proposed two different materials: 1) a composite of silicone and starch in different ratios, and 2) a dental impression material (vinyl polysiloxane). These materials can be modelled to the desired geometry, allowing the production of accurate and patient-specific physical replicas. The models developed were tested and evaluated by three expert microtia surgeons in a double-blind study considering parameters such as texture, firmness, and carving. It was found that the silicone starch composite showed mechanical characteristics very close to actual human cartilages such as pliability. Furthermore, the price is very low (0.60 €). The materials used in the last three studies enable the use of manufacturing methods that allow to obtain accurate geometries of the costal cartilage leading to a more realistic and effective simulation.

On the basis of the reported literature, it is possible to draft some practical considerations on the different materials tested to date for the AER reconstruction. Table 1 shows all the materials used to simulate human coastal cartilage, highlighting the advantages and disadvantages of each of them. From the reported analysis it is evident that silicone-based trainers can today be considered the best option to faithfully reproduce the ear anatomy.

## 4. Methods to Simulate Ear Reconstruction

The availability of materials used to create physical ear replicas to plan AER intervention has led at the same time to the development of several different methods to create simulators able to aid the surgeon to practice safely this complex operation. Almost all scientific works dealing with this topic share two fundamental components to be considered:

- The costal cartilage, i.e., the component from which to extract the basic material for the realization of the ear framework;
- The reference ear, i.e., the template to copy to obtain the ideal ear.

For both aspects, thanks to the technological evolution and in particular to the increased availability and precision of image acquisition and 3D printing technologies, it has been possible to obtain most accurate and immersive trainers, and, above all, patient-specific models for preoperative planning.

### 4.1. Costal Cartilage Fabrication Methods

An ideal simulator would deliver an accurate copy of the harvested patient cartilage to be used to replicate the volumes, the shapes, and therefore, the geometry of the ear regions. In fact, one of the objectives of the simulator is to train the surgeon to identify in the available costal cartilage where to carve out the components of the framework of the ear defining the best cutting strategy for an optimization of the available cartilage stock.

Thanks to the wide versatility of silicones in simulation coupled with 3D printing technologies, it possible to obtain 3D physical models able to accurately replicate any anatomical geometry.

The simplest method, as proposed in Reference [59], is to acquire shapes by imprinting actual costal cartilage. This allows to obtain a negative copy of the anatomy that is used as a mold to produce the positive copy by pouring the silicone inside. Once the silicone cures inside the mold, it can be extracted and used for the simulation of the surgery. With this method it is possible to obtain one of the two components of a generic simulator of the AER, useful for educational training of beginners, but not for preoperative simulation. In fact, the application of this method requires the handling of the actual cartilage of the patient and this is possible only inside the surgery room. For the training of physicians, it is sufficient to have accurate replicas of the costal cartilages of a generic patient, which allows to repeat the procedure of ear reconstruction in the same initial conditions achieving an effective educational training through repetitive performances, the evaluation of them, and the correction of eventual mistakes.

**Figure 4:**
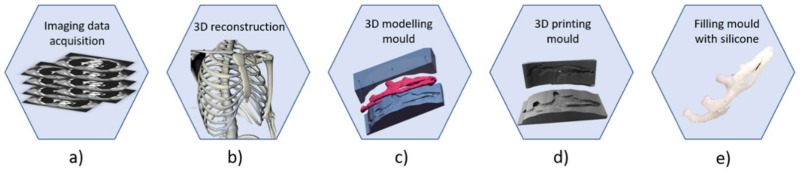
Workflow to produce an accurate replica of costal cartilage: **a**) image data acquisition of the thoracic region through with CT scan; **b**) image processing and 3D reconstruction of ROI (Region Of Interest); **c**) modelling of mold with 3D modelling software; **d**) printing of the mold; **e**) pouring silicone rubbers inside the mold.

In recent years, thanks to the implementation and combination of diagnostic imaging techniques and advanced technologies including reverse engineering (RE), computer-aided design (CAD), and additive manufacturing (AM), new methods have been used to produce accurate replicas of human anatomy. More specifically, in the case of AER the great potential of using these technologies lies in the possibility of obtaining very precise and specific geometries of the patient’s costal cartilage. This results in the production of simulators that can no longer be used just for training with a “general” geometry, but also for a realistic preoperative simulation with a “custom” geometry [61,62]. Figure 4 shows the steps necessary for the production of the replica of the costal cartilage. The first step consists in the image data acquisition through a high-resolution computed tomography of the thoracic region of the subject (Figure 4a). The diagnostic images in DICOM format are then imported into dedicated post-processing software such as 3D Slicer (Surgical Planning Laboratory, Brigham and Women’s Hospital, Harvard Medical School, Boston, MA, USA) or Mimics (Mimics® Innovation Suite, Materialise NV, Leuven, Belgium). The data are processed to reduce imaging noise, isolate the anatomy of interest, smooth the surface of the anatomy, and obtain the 3D computer model of the patient-specific costal cartilage (Figure 4b). A 3D mesh model, in STL (STereo Lithography interface )format, is exported to 3D modeling software such as Rhinoceros (https://www.rhino3d.com). The negative model is constructed through the Boolean mesh of the imported positive model (Figure 4c), then it is sent to a 3D printer that produces the physical mold by depositing layer-by-layer the plastic material (Figure 4d). Finally, the positive copy of the costal cartilage is obtained by filling the mold with selected silicone rubbers with mechanical characteristics such as hardness, elasticity, and textures as similar as possible to those of the costal cartilage (Figure 4e).

The adoption of this method to produce cartilage replicas allows to obtain an accurate copy of the anatomy of the patient to be operated, ensuring a proper planning and realistic simulation of AER, allowing the surgeon to observe the volumes of cartilage available before entering in the surgery room, and thus identify the ideal anatomical site from which to extract the elements of the ear to be reconstructed. Therefore, the most important and revolutionary contribution to the improvement of ear reconstruction training obtained through RE, CAD, and AM technologies is the possibility to identify the best strategy to obtain the anatomical elements from the cartilages minimizing the amount to cut; this is possible thanks to a quite real reproduction of the rib cartilages’ volume and geometry.

### 4.2. Reference Ear and Tools Fabrication Methods

The second aspect to be taken into account to create a trainer to help surgeons in performing the AER consists in the creation of a physical replica of the ear to be reconstructed. Such a replica can, in fact, be used as a reference during AER intervention since it is based on the actual shape and size of the contralateral ear. By shaping the patient ear following the simulated ear framework it is ideally possible to obtain an ear identical to the healthy one and with a natural aesthetic appearance.

**Figure 5:**
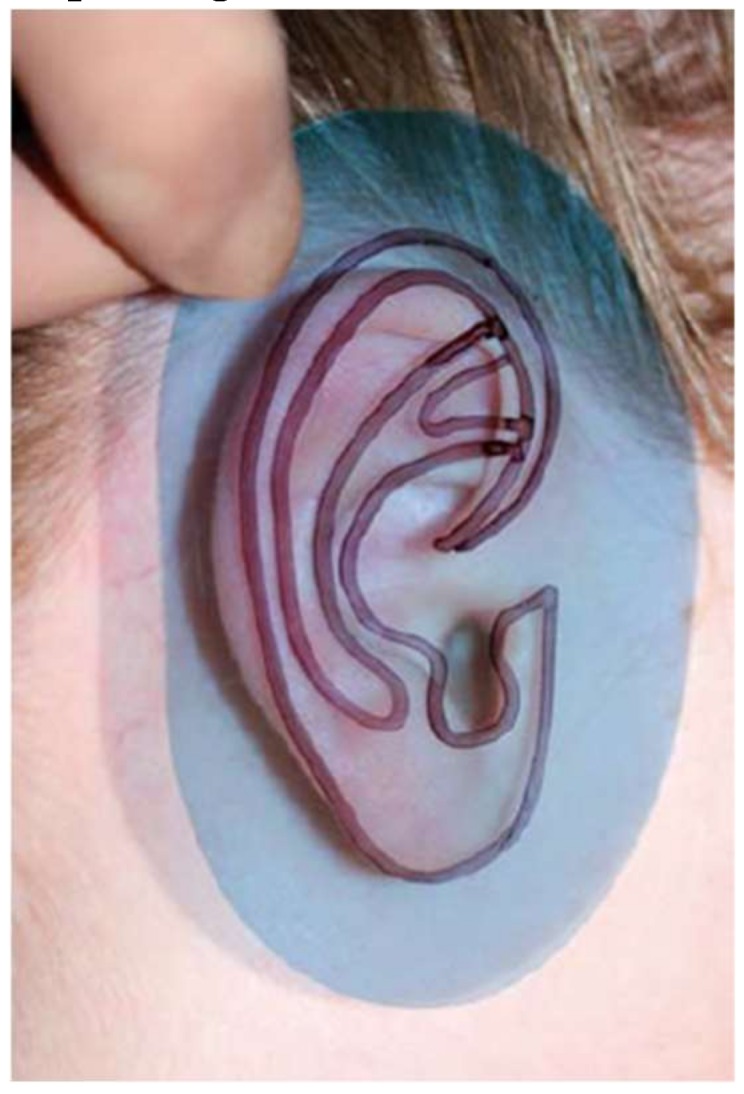
2D template of the ear. A translucent X-ray film is placed against the opposite ear and the main’s shape features are hand-traced, creating a 2D projection of the 3D shape.

The traditional approach to create the reference ear involves the creation of a 2D template using translucent X-ray film which is placed against the normal ear, and its shape features are hand-traced, creating a 2D projection of the 3D shape (Figure 5).

Such a 2D template is used by the surgeon as a visual guide during the cutting, modelling, and carving of the costal cartilage. However, this approach has several limitations; first of all, the X-ray film-based acquisition is affected by an error due to the pressure that is exerted on the ear while the silhouette is traced. Such a pressure, in fact, causes a deformation of the external ear, thus leading to an inaccurate drawing of the structure [63]. Furthermore, the 2D template does not convey the necessary information about the three-dimensional geometry of the ear, i.e., height, thickness, depth features of the anatomical elements of the ear structure (helix, antihelix, tragus–antitragus, concha, and scapha [64]). More in detail, while the ear is characterized by over 14 three-dimensional structures, through the 2D template only 6 to 8 of them can be captured, and systematic errors arise from the transformation of the actual 3D ear anatomy structure into a 2D simplified structure. Consequently, during the procedure, the physician is forced to find the missing information directly on the patient, moving back and forth from the work table on which he reconstructs the structure of the ear to the operating table, directly observing the *healthy* part, capturing the 3D shape of the contralateral ear, and mentally mirroring it to obtain the three-dimensional geometry of the ear to be reconstructed. As a result, operating times are longer, health costs increase, and the risk of infection for the patient is greater. In the case of the training or the preoperative simulation, the patient cannot be asked to be present during the surgeon’s training which is necessary to reconstruct the ear or to simulate the patient-specific surgery. On the other hand, the simulation of the reconstruction without three-dimensional information is incomplete and may lead to unsatisfactory aesthetic results.

To address these limits a 3D template must be fabricated which provides detailed 3D information and features of the ear’s structure overcoming the necessity of the continuous presence of the patient during the simulation of AER. Reverse Engineering CAD modelling, and AM technologies are able to meet this need fabricating three-dimensional template and overcoming the difficult and imprecise procedure of 2D line drawing on the ear structure.

**Figure 6:**
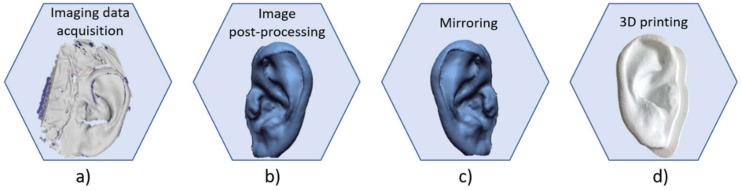
Workflow to produce three-dimensional ear template: (**a**) image data acquisition of the healthy ear; (**b**) image post-processing and digital 3D reconstruction; (**c**) mirroring of the ear; (**d**) 3D printing of the model.

Figure 6 shows the main steps for the construction of a three-dimensional ear-shaped template. The first step consists in the imaging acquisition of the healthy ear of the subject with a high accuracy able to capture the detailed features of the auricular structure (Figure 6a).

As with costal cartilage, diagnostic imaging techniques such as CT or MRI can be employed [14,16,20]. The tendency of clinicians is to use diagnostic imaging techniques; however, these have some disadvantages such as high cost, patient exposure to radiation in the case of computed tomography, and the large size and inability to transport medical equipment. Therefore, when only external ear shape is needed, an appropriate method is to use three-dimensional surface scanning or photogrammetry techniques, which are cheaper than medical imaging and mainly radiation free. The second step consists in isolating from acquired 3D data the ROI which is processed using typical RE operations such as hole filling, edge smoothing, and filtering to remove artefacts with the final aim of improving the quality of the 3D model surfaces (Figure 6b). Then, the processed model is mirrored to obtain the ear to be reconstructed (Figure 6c). Finally, the digital model is exported in the STL format prior to being 3D printed using the abovementioned framework (Figure 6d).

**Figure 7:**
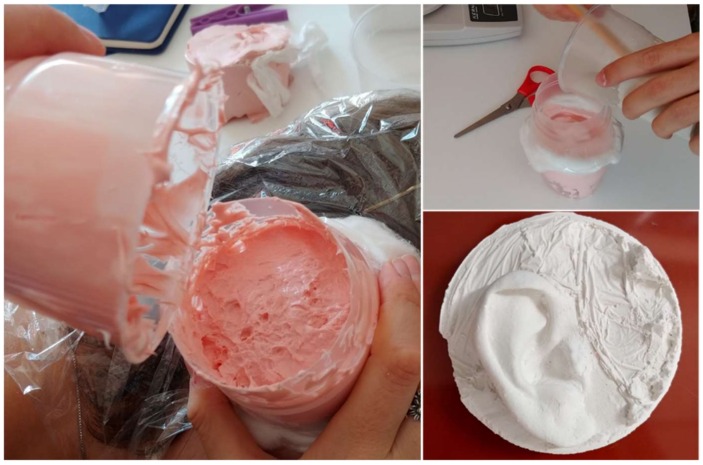
Acquisition of the shape of the ear by means of impression material.

Byoungjun et al. [64] carried out a study on seven children aged 11 to 16 suffering from microtia with the aim of studying and improving the aesthetic results of AER by giving a surgeon patient-specific three-dimensional models of their ears. For children it is often difficult to stand still for the time necessary for the scanning process; consequently, they created an accurate physical replica of the healthy ear by implementing casting-based technique. More specifically, they made a mold of the healthy ear of each patient by pouring an alginate impression material into a frame placed around the ear to capture. Then, they filled the mold with dental stone creating a cast of the patient’s normal ear (see Figure 7).

A 3D laser scanner (3D scanner HD, NextEngine, 2995 €) was then used to obtain the 3D CAD model from the dental stone copy. To ensure good model resolution, the 2D images were collected at intervals of 30°. The digital model was then mirrored and additive manufactured. A comparison in terms of accuracy between the traditional 2D template and the 3D template of the ear with the casted ear models showed that the average percentage differences were very different; more specifically the difference between the 2D template and the casted ear were 16.03% on average, while the difference between the 3D template and the casted ear were 2.31% on average. Therefore, the 3D model was a much more precise reference for surgeons during the simulation of the AER operation and the operation itself.

Zhou et al. [63] used a 3D scanner (Artec Spider, Artec Group, 11,000 €) with a resolution of 0.1 mm to obtain the auricular structure of 40 subjects affected by microtia. Data were processed using a Reverse Engineering CAD software (Geomagic Studio, 3D Systems, Rock Hill, SC, USA) and two different templates of the affected ear were designed and printed: a 2D sheet ear-shaped mold and a 3D template. The two models, after being properly sterilized, were used to assist surgeons during the AER of 40 subjects. The outcomes of surgery were compared to the results obtained on 20 other subjects using the traditional X-ray film. The comparison showed the highest accuracy of the results were obtained with the novel templates in terms of symmetry, length, width, cranioauricular angle and substructures of the reconstructed ear. In addition, surgical times were reduced by about 15 minutes compared to the method assisted by X-ray film template. The benefits of using the three-dimensional model were also confirmed by a double-blind study [7] that compared the aesthetic clinical results of AER performed with the help of the traditional 2D template with the ones obtained with the use of three-dimensional templates. Both the doctors’ evaluations and the patients’ satisfaction were surveyed, taking into consideration parameters such as appearance, shape, size, and similarity. It emerged that the group treated using 3D models as a training set obtained higher scores from both physicians and patients.

**Figure 8:**
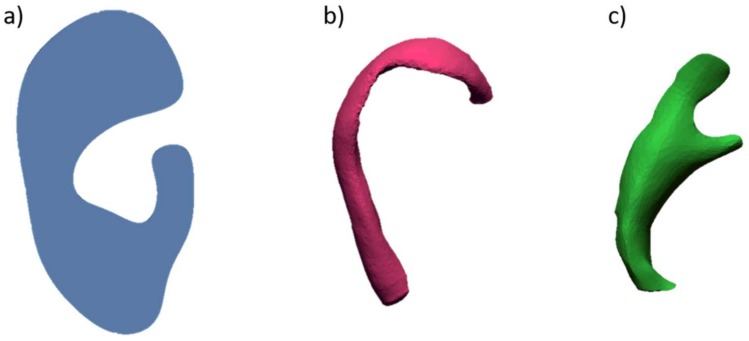
Anatomical elements: (**a**) Pinna (anti-helix, auricular tubercle, scaphoid fossa, and helix), (**b**) helix, (**c**) anti-helix.

In 2017, Flores et al. [65] introduced additional elements to assist the surgeon during the cutting and modelling of the costal cartilages. They acquired the unaffected ear of a patient with a high-resolution 3D digital photograph (3DMD, Atlanta, GA, USA, 6000 €), with a linear accuracy range of 0.5 mm or better. Captured data, in STL format, were processed using the modelling software Blender™ to sculpt and refine the structure of the digital auricular model. Then, the anatomical elements making up the earcup (helix, anti-helix and pinna) were isolated and extracted from the ear model (see Figure 8) using the Blender tools.

**Figure 9:**
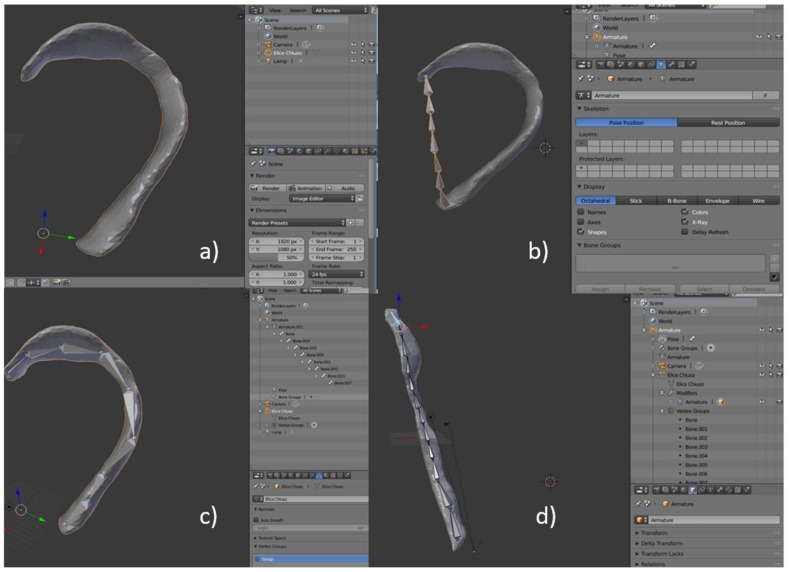
Process to unroll helix. (**a**) Import of the STL file in Blender; (**b**) creation of a bone chain; (**c**) placement of the bones within the helix; (**d**) digital straightening of the helix.

Subsequently, an unrolled version of the helix was obtained by inserting a digital armature and deforming it until a linear structure was obtained (Figure 9).

The digital models were finally printed in PLA with an FDM (Fused Deposition Modelling) printer (Builder Premium 3D Printer, Noordwijkerhout, The Netherlands, 3000 €), sterilized, and used in the surgery room, placing them on top of the costal cartilages as a reference for cutting and modelling.

The actual effectiveness of this method has yet to be demonstrated by the authors as it has been tested only on a patient whose pre- and postoperative image was reported in the related paper. A comparative study would be needed to compare the improvements of the ear reconstruction in terms of, for example, surgical times and aesthetic results with and without the anatomical models designed by the authors. However, it is reasonable to think that good outcomes can be achieved as the new models are an additional guide compared to the three-dimensional template seen above.

Bos et al. [66] developed a parametric ear model usable as template for modelling during ear reconstruction simulation. They have considered that the anatomical element is characterized by several features that are common to all individuals. Based on this observation and the CT scans of four human cadaver’s ear, they created a basic model of the ear using Rhinoceros software. The model has been made parameterizable, thanks to a plug-in of the Grasshopper software, enabling the modification of dimensions and the fitting on of different patient-specific shapes and dimensions.

In Table 2, the data relating to the production costs of the ear reconstruction simulator considering the raw cost of materials and manpower adopting the different manufacturing methods described above are reported. A column is dedicated to the list of software and machinery that a laboratory must have to employ the specific manufacturing method.

## 5. Methods to Evaluate a Surgeon’s Performance

**Figure 10:**
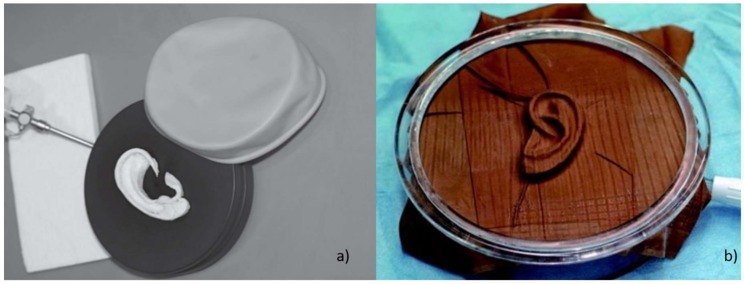
Devices to simulate the skin over the ear framework. (**a**) “Trainer” by Stortz (Tuttlingen, Germany) to simulate the skin over the reconstructed three-dimensional ear framework [28]; (**b**) Similar trainer developed by Oyama et al. [67].

Simulation allows surgeons to familiarize themselves with the complex procedure of reconstruction and to study and optimize the cutting of cartilage outside the surgery room, thus anticipating this phase (preoperative planning) and reducing the operating time. However, the aesthetics pleasantness of the ear reconstructed during simulation must be qualitatively assessed, this aspect being an important requirement for the patient undergoing surgery. In particular, it is important to assess the expected aesthetic impact obtainable when the framework is placed in the skin pocket. For this purpose, Firmin et al. [28] proposed the device (Stortz, Tuttlingen, Germany) shown in Figure 10a. Such a device allows to observe the quality of carving by placing the three-dimensional framework under a silicone sheet that simulates the skin. A drainage suction eliminates the air below, allowing the sheet to adhere to the framework. In this way, it is possible to reliably simulate the actual positioning of the reconstructed ear in the skin pocket. In fact, the relief and contours visible when the three-dimensional framework is inserted into the skin pocket are aesthetically very similar to the ones obtained using the proposed device.

A very similar system was designed by Oyama et al. [67]. The device (Figure 10b) allows to improve the precision and the quality of the aesthetic results of AER and can be used in the surgery room to simulate the insertion of the framework and to check the result of the reconstructed ear several times until a satisfactory result is reached.

## 6. Conclusions

Autologous Ear Reconstruction is among the most challenging surgical procedures encountered by reconstructive surgeons. To obtain an ear with a realistic 3D configuration almost perfectly symmetrical to the healthy contralateral ear, high manual skills are required. In addition, the structure of the ear and malformation is unique for each patient; therefore, a careful consideration of the individual patient and the most suitable surgical approach is crucial. In this context, the importance of improving surgeons’ training, and at the same time, to carry out a consistent preoperative planning before entering the surgery room is vital. The use of RE and AM technologies allows surgeons both to repeatedly train on an accurate geometry obtained from actual data and to plan and simulate a specific AER on the patient-specific geometry. For this reason, the application of RE and AM technologies have a significant impact on the results of the AER, improving the technical capabilities of the surgeons involved and therefore the clinical results of the surgery. In literature, different studies were carried out on the best materials and methodologies to give the reconstructive surgeons a tool to practice and simulate AER surgery. The present work has described in a systematic way the history of materials adopted and the evolution of the methodologies employed to produce accurate physical replicas of costal cartilage and the reference ear. Future works will be addressed to the research and the study of new methods to improve the simulation and surgeon’s education to AER surgery, providing tools able to guarantee a higher assurance of the aesthetic reconstruction outcomes less linked to artistic and technical skills of the clinician, and decrease time in ear framework fabrication.

## Figures and Tables

**Table 1:** Advantages and disadvantages of the materials used to resemble human costal cartilages.

Material	Advantages	Disadvantages	Price
Soap bar [50]	Readily available on the market Low cost	Different mechanical properties compared to human costal cartilage (elasticity, flexibility) Different shape that mismatches to human costal cartilages	1.20 €
Vegetables [51,52]	Readily available on the market Low cost Good consistency, flexibility, elasticity	Brittle Hard to carve Not elastic Different shape and amount	0.20 €
Animals [53,54]	Easily available Low cost Good consistency, flexibility Good replica of anatomy of the human cartilage shape	Specific storage conditions Risk of disease transmission Ethical problems	5.00 €
Human cadavers [55]	Accurate replica of the human cartilage shape	Limited availability Specific storage conditions Risk of disease transmission Ethical problems Potential calcification of tissues	2000 €
Plastic eraser [57]	Faithful texture No risk of disease transmission No ethical problems	Less elasticity Not suitable to 5-0 nylon sutures Inaccurate reproduction of geometries	4.00 €
Acrylic polyurethane [60]	Good cutting behavior Inexpensive No risk of transmission	Low elasticity compared to human cartilage	1.00 €
Silicone [58,59,61]	Fairly faithful reproduction of the geometries Faithful texture No risk of disease transmission No ethical problems Inexpensive		0.60 €

**Table 2:** Simulator fabrication costs (raw cost and manpower) of each method described.

Components	Fabrication Methods	Facilities and Software	Material	Manpower
Costal cartilages	Molding cartilage and pouring [59]	0.00 €	Silicone rubber: 13.00 €	2 h (professional surgeon)
	Imaging data segmentation 3D modelling mold 3D printing mold Pouring silicone rubber [61,62]	3D segmentation software (i.e., 3D Slicer): freeware 3D Modelling software (i.e., Rhinoceros): 995.00 € FDM desktop printer: 2500 €	Silicone rubber: 0.45 €–52.00 €	1 h (professional radiologist) 2 h (engineer) 10 h (printing time)
Reference ear and tools	Hand-tracing ear contour	0,00 €	Tracing paper 0.20 € or X-ray film 3.00 €	5 min (professional surgeon)
	Casting actual ear and pouring dental stoner Image acquisition of ear’s replica 3D modelling 3D printing of template [64]	3D laser scanner (i.e., 3D Scanner HD, NextEngine): 2995 € FDM desktop printer: 2500 €	6.00 €	4.5 h (engineer)
	Ear image acquisition with 3D scanner Data processing 3D printing template [63]	3D scanner (i.e., Artec Spider): 11,000 € 3D modelling software (Geomagic Studio): 9000 € FDM printer: 2500 €	0.50 €	4 h (engineer)
	Ear image acquisition with 3D digital photograph 3D modelling 3D printing [65]	3D scanner (3DMD): 6000 € FDM desktop printer (i.e., Builder Premium 3D): 3000 €	1.00 €	6 h (engineer)
